# Author's Reply

**DOI:** 10.18502/jovr.v15i1.5967

**Published:** 2020-02-02

**Authors:** Neha Goel

**Affiliations:** ^1^ICARE Eye Hospital and Postgraduate Institute, Noida, Uttar Pradesh, India

I thank Dr. Mahmood Dhahir Al-Mendalawi for his interest in our article and his comments. The patient described in this report was HIV negative.

